# A Cross-Sectional Study on the Prevalence and Risk Factors of Steroid-Induced Glaucoma at a Tertiary Eye Care Hospital

**DOI:** 10.7759/cureus.104759

**Published:** 2026-03-06

**Authors:** Kalyani VKS, Kalyani Malasane, Vidya Chelerkar, Prachi N Bakare, Rahul Deshpande

**Affiliations:** 1 Glaucoma, H.V. Desai Eye Hospital, Poona Blind Men's Association (PBMA), Pune, IND; 2 Ophthalmology, H.V. Desai Eye Hospital, Poona Blind Men's Association (PBMA), Pune, IND; 3 Ophthalmology, Postgraduate Institute and Yashwantrao Chavan Memorial (YCM) Hospital, Pimpri Chinchwad Municipal Corporation (PCMC), Pune, IND

**Keywords:** corticosteroids, intraocular pressure, ocular hypertension, risk factors, steroid-induced glaucoma

## Abstract

Background: Steroid-induced glaucoma (SIG) is a well-recognized complication of corticosteroid therapy, leading to elevated intraocular pressure (IOP) and potential irreversible optic nerve damage. Despite widespread steroid use for ocular conditions, inadequate monitoring, especially with over-the-counter (OTC) preparations, increases the risk of SIG. This study aimed to determine the prevalence and identify the risk factors associated with SIG in a tertiary eye care setting.

Methods: This cross-sectional study was conducted at the Glaucoma Department of Poona Blind Men's Association (PBMA)'s H.V. Desai Eye Hospital in Pune, India, over 20 months. A total of 190 patients (380 eyes) with SIG were identified from 4609 glaucoma cases. Inclusion criteria involved patients aged >10 years with a history of ocular steroid use and newly diagnosed SIG. Examinations included visual acuity, IOP measurement, gonioscopy, optic nerve evaluation, perimetry, and imaging. Patients were treated with anti-glaucoma medications (AGMs) or surgery when indicated. Data were analyzed using IBM SPSS Statistics for Windows, Version 23.0 (IBM Corp., Armonk, New York, United States).

Results: SIG prevalence was 4.1%. Most patients (59%) were above 45 years; 53.2% were male. While 60.5% had no known risk factors, 13.1% had diabetes, 12.1% were under 20 years, and others had high myopia or rheumatoid arthritis. Topical dexamethasone was the most used steroid (50%), with most using steroids for 1-3 months. IOP significantly reduced from a mean of 26.8 mmHg at baseline to 14.1 mmHg at six months (p<0.001). AGM sufficed in 93.6% of eyes, while 6.4% required trabeculectomy. Visual acuity and optic disc status remained largely stable.

Conclusion: SIG is a preventable cause of vision loss. Early detection, rational steroid use, regular IOP monitoring, and public education are essential to mitigate this iatrogenic burden.

## Introduction

Steroid-induced ocular hypertension and steroid-induced glaucoma (SIG) are well-recognized adverse effects of corticosteroid therapy, characterized by elevated intraocular pressure (IOP) and subsequent glaucomatous optic nerve damage. The association between corticosteroid use and raised IOP was first identified in the 1950s by Gordon et al., who observed this phenomenon following the systemic administration of adrenocorticotropic hormone (ACTH) [1]. Subsequently, similar effects were noted with topically administered corticosteroids [2].

Corticosteroids exert multiple physiological effects on the trabecular meshwork (TM), the primary site of aqueous humor outflow. These include thickening of trabecular beams, reduction in intertrabecular spaces, accumulation of extracellular matrix, and cellular remodeling, all of which contribute to increased resistance to aqueous outflow and IOP elevation. These changes are more prominent in individuals known as "steroid responders", who can be identified by significant IOP elevation following steroid use [3,4].

Approximately 5-30% of the general population exhibit a steroid response, with greater susceptibility among individuals with primary open-angle glaucoma (POAG), ocular hypertension, or a family history of glaucoma [3,5]. The likelihood and magnitude of this response are influenced by the route of administration, the potency and duration of steroid therapy, and the underlying ocular condition. While topical corticosteroids are the most common culprits, intravitreal, periocular, and even dermatologic formulations have also been implicated in IOP elevation [6,7].

Despite the widespread use of corticosteroids in ophthalmic practice, especially for conditions such as allergic conjunctivitis, vernal keratoconjunctivitis (VKC), and post-surgical inflammation, routine monitoring of IOP is often overlooked, particularly in cases of unsupervised or over-the-counter (OTC) use. Unmonitored or prolonged use can lead to irreversible optic nerve damage and permanent visual impairment [8]. Given the preventable nature of SIG with timely diagnosis and appropriate intervention, this study was undertaken to estimate the prevalence of SIG and identify the associated demographic and clinical risk factors among patients attending a tertiary eye care hospital in Western India.

## Materials and methods

This hospital-based cross-sectional observational study was conducted in the Glaucoma Department of Poona Blind Men's Association (PBMA)'s H.V. Desai Eye Hospital in Pune, India, over 20 months (October 2020 to June 2021), after obtaining approval from the institute's Institutional Ethics Committee (approval number: HVDEH/IEC/2066101118). The study aimed to determine the prevalence and identify the risk factors associated with SIG. A total of 190 patients were enrolled, based on a sample size calculated using a 95% confidence level, an 80% power, and a 3% absolute precision. Patients aged over 10 years with newly diagnosed SIG and a history of ocular steroid use for conditions like VKC, diabetic macular edema, or post-cataract surgery were included. Patients with POAG, uveitis, systemic immunosuppression, or eventful cataract surgeries were excluded.

Diagnosis of SIG was based on IOP ≥22 mmHg on two consecutive visits and/or glaucomatous optic disc damage. Informed consent was obtained from all participants. Clinical data collected included demographic profile, indication and duration of steroid use, mode of administration, steroid type and concentration, family history of glaucoma, systemic conditions, and previous ocular history. All patients underwent thorough ophthalmic evaluation, including best-corrected visual acuity (BCVA), slit-lamp examination, IOP measurement by Goldmann applanation tonometry, pachymetry, gonioscopy using Goldmann and Zeiss lenses, optic nerve evaluation with +90D lens, and visual field analysis using the Humphrey field analyzer with SITA 24-2 protocol.

Steroids were discontinued where not medically essential, and anti-glaucoma medications (AGMs) were initiated based on IOP and clinical indication. Follow-up was conducted at one, three, and six months, with additional visits for patients with high baseline IOP. If IOP was <40 mmHg, patients received oral acetazolamide and topical medications; if it was ≥40 mmHg, syrup glycerol was also added. In cases where IOP remained uncontrolled despite maximum medical therapy, trabeculectomy with mitomycin-C (0.02% for three minutes) was performed. Treatment decisions were revised based on IOP trends and optic nerve findings at follow-up visits.

The collected data were entered in Microsoft Excel (Microsoft Corporation, Redmond, Washington, United States) and analyzed using IBM SPSS Statistics for Windows, Version 23.0 (IBM Corp., Armonk, New York, United States). Descriptive statistics summarized key variables, while chi-squared/Fisher's exact tests and logistic regression identified significant associations. A p-value of <0.05 was considered statistically significant.

## Results

Out of 4,609 glaucoma cases presenting to the Glaucoma Department of PBMA's H.V. Desai Eye Hospital over a 12-month period, 190 patients (4.1%) were diagnosed with SIG. These 190 patients contributed a total of 380 affected eyes. Among the study population, 56 patients (29.6%) were aged over 60 years, while 55 patients (29.1%) were between 45 and 60 years of age, indicating that nearly 59% of the participants were above 45 years. In contrast, only 13 patients (6.9%) were under 15 years of age. The mean age of the study population was 46.92 years, with a median age of 50 years. Of the 190 participants, 101 (53.16%) were male and 89 (46.84%) were female (Table 1).

**Table 1:** Age and gender distribution of patients with steroid-induced glaucoma (N=190)

Parameter		Number of patients	Percentage (%)
Age (years)	<15	13	6.84%
	15-30	33	17.37%
	30-45	32	16.84%
	45-60	55	28.95%
	>60	57	30%
Gender	Male	101	53.16%
	Female	89	46.84%

Among the 190 patients diagnosed with SIG, approximately 60% had no identifiable predisposing risk factors. However, among the remaining 40% of patients, diabetes mellitus was the most common associated condition, observed in 13% of the cases. A total of 12.1% of the patients were below 20 years of age, highlighting young age as a potential risk factor. Additionally, 4.8% had a positive family history of glaucoma, 4.2% had high myopia, and 5.2% were known cases of rheumatoid arthritis not receiving systemic steroid therapy (Table 2).

**Table 2:** Risk factors identified among patients with steroid-induced glaucoma (N=190)

Risk factor	Number of patients	Percentage (%)
Diabetes mellitus	25	13.1%
Age <20 years	23	12.1%
Family history of glaucoma	9	4.8%
High myopia	8	4.2%
Rheumatoid arthritis	10	5.2%
No risk factors	115	60.5%

The mean IOP showed a significant and consistent decline over time following the withdrawal of steroids and initiation of anti-glaucoma treatment. At the first visit, the mean IOP was 26.84 mmHg (SD=7.13), which reduced substantially to 16.29 mmHg at one month, 14.57 mmHg at three months, and 14.07 mmHg at six months. The difference in mean IOP across these time points was statistically significant, as indicated by the ANOVA result (F=523.132; p<0.001) (Table 3).

**Table 3:** Mean intraocular pressure (mmHg) at different time intervals (N=190)

Time point	Mean intraocular pressure	SD	Min	Max	F-stat	P-value
First visit	26.84	7.13	10	54	523.132	<0.001
One month	16.29	5.24	8	40		
Three months	14.57	3.81	6	32		
Six months	14.07	3.11	6	26		

Multiple comparisons further confirmed significant reductions between all time points. The mean difference in IOP between the first visit and one month was 10.547 mmHg (p<0.001) and between the first visit and three months was 12.268 mmHg (p<0.001). A slightly greater reduction of 12.764 mmHg was observed between the first visit and six months (p<0.001). Smaller yet statistically significant decreases were noted between one and three months (1.721 mmHg; p<0.001) and between one and six months (2.217 mmHg; p<0.001). However, the difference between three and six months (0.498 mmHg) was not statistically significant (p>0.05), indicating that IOP had largely stabilized by the third month of treatment (Table 4).

**Table 4:** Intercomparison of intraocular pressure at different time intervals

Period	Period	Mean difference	P-value
First visit	One month	10.547	<0.001
	Three months	12.268	<0.001
	Six months	12.764	<0.001
One month	Three months	1.721	<0.001
	Six months	2.217	<0.001
Three months	Six months	0.498	>0.05

Visual acuity remained largely stable over the follow-up period. At baseline, 67.2% of eyes had good vision (6/6-6/9), which remained consistent at around 66% through six months. Mild and moderate visual impairment levels showed minimal variation. A slight increase in severe visual impairment (6/60) was noted, from 3.4% to 4.6%. Overall, no significant change in vision was observed, indicating effective control of IOP without major visual deterioration (Table 5).

**Table 5:** Comparison of visual acuity at different time intervals (N=378 eyes)

Visual acuity	First visit	One month	Three months	Six months
6/6-6/9	254 (67.2%)	247 (66.6%)	243 (66.2%)	244 (66.5%)
6/12-6/18	79 (20.9%)	80 (21.6%)	82 (22.3%)	79 (21.5%)
6/24-6/36	12 (3.2%)	14 (3.8%)	10 (2.7%)	11 (3%)
6/60	13 (3.4%)	12 (3.2%)	16 (4.4%)	17 (4.6%)
Others	20 (5.3%)	18 (4.9%)	16 (4.4%)	16 (4.4%)

The mean cup-to-disc ratio (CDR) showed minimal change over the study period. At the first visit, the mean CDR was 0.4565, which slightly increased to 0.4594 at one month and 0.466 at six months. However, this increase was not statistically significant (F=0.207; p=0.813), indicating no meaningful progression of optic nerve cupping during follow-up. This suggests that with appropriate management, structural optic nerve damage was effectively controlled in most patients (Table 6).

**Table 6:** Cup-to-disc ratio at different time points

	N	Mean	SD	Min	Max	F-stat	P-value
First visit	376	0.4565	0.20243	0	0.95	0.207	0.813
One month	371	0.4594	0.20721	0	0.95		
Six months	363	0.466	0.20242	0	0.95		

Perimetry was performed for all patients diagnosed with SIG, with 82.6% showing normal visual fields that suggest early detection and effective management. Advanced field loss was seen in 3.9% of patients, while isolated defects and various types of arcuate scotomas accounted for 5.2% and 5.5% of cases, respectively, with a minor 1% demonstrating learning effects. Among the 340 eyes treated with steroids, 33.2% received treatment for 1-3 months and 31.2% for 3-6 months, with a scant 3.5% using steroids for over 12 months, including two children with VKC who suffered severe glaucomatous damage. Eye drops were the predominant mode of administration (95.3%), compared to eye ointments (3.5%) and other methods, making up less than 1.5% overall. Dexamethasone was the steroid used in 50% of cases, followed by prednisolone in 31.4%, with loteprednol, betamethasone, and fluorometholone comprising the remainder at 7.3%, 6.7%, and 4.4%, respectively.

Steroids were most commonly used for OTC symptoms such as redness and watering (24%), followed by VKC in 19% and allergic conjunctivitis in 12.5% of cases. Postoperative indications like post-cataract surgery (12.9%) and post-implantable phakic contact lens (post-IPCL) implantation (4.2%) were also notable. A variety of other less common clinical conditions, including episcleritis, chemical injuries, graft rejection, and post-surgical inflammation, collectively accounted for the remaining cases.

In 93.6% of cases (318 eyes), AGMs were initiated. Patients using OTC steroid drops for allergic eye conditions were evaluated and counseled about the potential side effects and advised to discontinue inappropriate use. Surgical intervention via trabeculectomy was performed in 22 eyes with uncontrolled IOP despite combination AGM therapy.

## Discussion

Corticosteroids are potent anti-inflammatory agents widely used in ophthalmology to treat various ocular inflammatory and allergic conditions and as a routine postoperative therapy following ocular procedures [1,2]. However, their use is associated with a well-documented risk of ocular hypertension and SIG, especially with topical forms. The present study aimed to assess the clinical profile, risk factors, duration of steroid use, and management outcomes in patients with SIG. In this study, among the 4609 glaucoma cases treated at the Glaucoma Department of PBMA's H.V. Desai Eye Hospital, 4.1% (190 subjects) were diagnosed with SIG, a finding consistent with previous studies. Gupta et al. reported a prevalence of 4.7% in children attending a glaucoma clinic [9], while Gadia et al. found SIG to constitute 8% of all secondary glaucomas [4]. However, the latter included systemic steroid use, whereas our study focused solely on ocular steroid administration.

The age distribution in our study showed that nearly 59% of subjects were older than 45 years, with only 6.9% under 15 years. While gender and racial differences are not strongly associated with SIG, our sample showed a slight male predominance (53.2%). The risk factor profile revealed that 40% of patients had identifiable risk factors, with diabetes mellitus being the most prevalent (13%). Becker et al. noted that diabetic patients exhibit greater IOP elevation in response to corticosteroids [10]. However, Park et al. contradicted this in their study on intravitreal triamcinolone, reporting no correlation between diabetes and IOP elevation [11].

A positive family history of glaucoma was noted in 4.8% of patients. Becker and Hahn demonstrated a significantly higher IOP response to steroids among offspring of POAG patients [3]. Additionally, 12.1% of our patients were under 20 years, with younger age being recognized as a risk factor for heightened steroid response and earlier peak IOP elevation, as shown by Lam et al. [12]. Myopia, particularly high myopia, was found in 4.2% of our cohort. While some studies associate high myopia with increased POAG risk [13], others do not find a significant correlation [11]. Connective tissue disorders such as rheumatoid arthritis were observed in 5.2% of cases. Gaston et al. reported a higher incidence of steroid responsiveness in this subgroup, suggesting a heightened risk of SIG [14].

The most frequent indication for steroid use was OTC prescriptions for nonspecific ocular symptoms (24%), followed by VKC (19%) and allergic conjunctivitis (12.5%). This differs from earlier studies in children where VKC was the predominant cause [15,16]. The widespread availability of potent steroids like dexamethasone OTC, due to low cost and rapid symptom relief, likely contributes to their misuse. In our study, dexamethasone was the most commonly used steroid (50%), followed by prednisolone (31.4%). Fluorometholone, considered less potent, was the least used (4.4%). The duration of steroid use is a key determinant of SIG risk. Most patients in our study had used steroids for 1-3 months. Among the few who used them for over 12 months, two children with VKC developed irreversible glaucomatous optic neuropathy. Similar findings were reported by Gupta et al., with dexamethasone being the primary offending agent [9].

The mode of steroid administration also plays a role in SIG development. Our findings align with previous literature that shows topical and intravitreal routes are more likely to cause elevated IOP [5]. While discontinuation of steroids can reverse IOP elevation in some cases, prolonged use may lead to permanent trabecular meshwork damage requiring long-term AGM or surgery [6]. In our study, 93.6% of eyes responded to AGMs, and 6.4% required surgical intervention in the form of trabeculectomy. This is comparable to the findings by Sihota et al., who reported a surgical intervention rate of approximately 26.5% in their cohort [17].

The strength of our study lies in its comprehensive inclusion of patients with various indications for ocular steroid use, rather than restricting to a single etiology such as VKC. The findings emphasize that steroid eye drops are the most abused OTC medication, with most patients presenting within 1-3 months of initiation. However, a limitation of this study is its exclusion of systemic steroid users. Widespread unmonitored use of topical steroids for common eye complaints, often without physician oversight, underscores the need for awareness at multiple levels. Educating patients, caregivers, and even general practitioners about the risks of steroid misuse, especially the potential for irreversible vision loss, is crucial. Children and their parents should be counseled, particularly regarding VKC management, and consideration should be given to switching to non-steroidal immunosuppressants in chronic cases. Routine IOP monitoring post-ocular surgeries, such as cataract or pterygium excision, should be institutionalized as a standard practice. Lastly, policy interventions must target the easy availability of steroids OTC. Regulatory enforcement, along with public awareness campaigns, would help curb preventable blindness due to iatrogenic glaucoma.

## Conclusions

This study estimated the prevalence, identified risk factors, and reviewed the management of SIG. SIG was found in 4.1% of all glaucoma cases in a tertiary care center. Common indications for steroid use included VKC and allergic conjunctivitis, with OTC steroid misuse being a major contributor. With timely withdrawal of steroids and appropriate IOP control, SIG can often be reversed. This highlights the need for regular monitoring and policy-level control over OTC steroid access.
